# Combined resistance to oxidative stress and reduced antenna size enhance light-to-biomass conversion efficiency in *Chlorella vulgaris* cultures

**DOI:** 10.1186/s13068-019-1566-9

**Published:** 2019-09-16

**Authors:** Luca Dall’Osto, Stefano Cazzaniga, Zeno Guardini, Simone Barera, Manuel Benedetti, Giuseppe Mannino, Massimo E. Maffei, Roberto Bassi

**Affiliations:** 1 Dipartimento di Biotecnologie, Università di Verona, Strada Le Grazie 15, 37134 Verona, Italy; 2 Dipartimento di Scienze della Vita e Biologia dei Sistemi, Unità di Fisiologia Vegetale, Università di Torino, Via Quarello 15/a, 10135 Turin, Italy

**Keywords:** Microalgae, Chloroplast, Biofuel, Excess light, Photoprotection, Singlet oxygen, Biomass

## Abstract

**Background:**

Microalgae are efficient producers of lipid-rich biomass, making them a key component in developing a sustainable energy source, and an alternative to fossil fuels. *Chlorella* species are of special interest because of their fast growth rate in photobioreactors. However, biological constraints still cast a significant gap between the high cost of biofuel and cheap oil, thus hampering perspective of producing CO_2_-neutral biofuels. A key issue is the inefficient use of light caused by its uneven distribution in the culture that generates photoinhibition of the surface-exposed cells and darkening of the inner layers. Efficient biofuel production, thus, requires domestication, including traits which reduce optical density of cultures and enhance photoprotection.

**Results:**

We applied two steps of mutagenesis and phenotypic selection to the microalga *Chlorella vulgaris*. First, a pale-green mutant (*PG*-*14*) was selected, with a 50% reduction of both chlorophyll content per cell and LHCII complement per PSII, with respect to WT. *PG*-*14* showed a 30% increased photon conversion into biomass efficiency vs. WT. A second step of mutagenesis of *PG*-*14*, followed by selection for higher tolerance to Rose Bengal, led to the isolation of pale-green genotypes, exhibiting higher resistance to singlet oxygen (strains *SOR*). Growth in photobioreactors under high light conditions showed an enhanced biomass production of *SOR* strains with respect to *PG*-*14*. When compared to WT strain, biomass yield of the *pale green *+* sor* genotype was enhanced by 68%.

**Conclusions:**

Domestication of microalgae like *Chlorella vulgaris,* by optimizing both light distribution and ROS resistance, yielded an enhanced carbon assimilation rate in photobioreactor.

**Electronic supplementary material:**

The online version of this article (10.1186/s13068-019-1566-9) contains supplementary material, which is available to authorized users.

## Background

The rapid burning of fossil fuels impacts on Earth’s climate making the search for carbon-neutral fuels solutions urgent. Liquid fuels derived from photosynthetic organisms represent a renewable alternative to fossil fuels and a source of sustainable energy [1, 2]. Mass cultures of microalgae in photobioreactors (PBRs) are a promising source of biomass for biofuel production on a large scale, due to the high productivity and lipid content, far exceeding the best crops [3–5]. In both microalgae and land plants, photosynthetic reactions are carried out by membrane supercomplexes and soluble enzymes [6]; yet, due to a simpler cellular structure, microalgae are far more efficient in converting solar energy into biomass. Moreover, when growing on marginal lands, algae do not compete with food crops for arable soils and a number of species can accumulate high level of lipids, up to over 50% of their dry biomass [7]. After oil extraction, the residual lipid-free biomass can be used as bio-stimulant and fertilizer or fermented to produce biogas [8]. Microalgae are also useful for wastewater bioremediation and CO_2_ mitigation because of their high capacity to recover nitrogen, phosphorus and heavy metals from industrial, municipal and agriculture wastes [9, 10].

Among microalgae species, members of the genus *Chlorella* gained importance as robust biomass accumulating strains, allowing for sustainable industrial productions of high-value products and biofuels [11]. Under high irradiance or nutrient (N, P) deficiency, *Chlorella* mass cultures increase their neutral lipid content [12] in the form of triacylglycerols, which serve as cellular storage molecules, thus making these organisms promising candidates for lipid-based biofuels production. Potential coupling of oil production with either wastewater bioremediation or CO_2_ abatement technologies to industrial applications may decrease the cost of biofuel production as well as provide significant environmental benefits [10].

Production of biofuels from microalgae, however, still suffers from limitations, hampering cost effectiveness. These include the costs for PBRs construction and management, water pumping and mixing, axenic practices for preventing contamination of monocultures, harvesting biomass and lipid extraction [5]. In addition, there are physiological limitations such as low efficiency of light use, especially under high irradiance. The maximal theoretical efficiency of photosynthetically active radiation (400–700 nm) (PAR) solar energy conversion into biomass is about 27% [13]. However, such values are only observed at low light intensity in laboratory-scale growth trials, while efficiency drops below 6% in outdoor cultures at full sunlight intensities [12]. Limits in biomass yield can be ascribed to a number of factors [14], including (i) light-saturation effect, (ii) inhomogeneous light distribution within a mass culture and (iii) photoinhibition.

The light-saturation effect becomes evident when considering the light response curves for photosynthesis compared with the rate of light absorption [15]. In low-light conditions, photosynthetic rates increase with increasing irradiance, and the rate of photon absorption is correlated with that of electron transport from water to CO_2_; at higher irradiance, the photosynthetic rate increases non-linearly with respect to light intensity, reaching light saturation (*P*_max_). Within saturation range, excess energy is dissipated into heat. At even higher fluency, net assimilation decreases due to oxidative photoinhibition.

Within the light limited range, energy is efficiently used for photosynthesis. When light irradiance overcomes the rate of downstream biochemical reactions, excess absorbed energy is wasted as heat, thus impairing light-to-biomass conversion efficiency [16]. Additional energy loss derives from the inhomogeneous light distribution in the algal culture. The high optical density of algal cells at the surface causes a steep light gradient which leaves inner layers below compensation point with respiration causing energy loss. The high Chl content per cell maximizes the photon capture in the natural environment, with limiting light and low cell density. To this aim, large arrays of antenna complexes (Light-harvesting complexes, LHCs), binding chlorophylls (Chl) and carotenoid (Car) in *quasi*-*molar* concentration, enhance exciton supply to reaction centers, hosting photochemical reactions [17]. However, large antenna systems do not enhance overall productivity in a PBR because the high optical density readily leads to saturation of photosynthesis in the surface layers, while the inner space becomes light limited. The resulting inhomogeneous light distribution impairs productivity [18]. Upon sustained over-excitation experienced by cells of surface layers, increase in lifetime of Chl singlet excited states (^1^Chl*) and intersystem crossing to the Chl triplet state (^3^Chl*) occur. Moreover, reaction with molecular oxygen (O_2_) yields singlet oxygen (^1^O_2_) hence photoinhibition of PSII, a complex highly susceptible to light damage [19, 20]. Algal cells rapidly shift between layers with low vs. high irradiance due to mixing, which impairs the light acclimation capacity of their photosynthetic apparatus.

Domesticating microalgae for enhanced growth rate in PBRs requires introduction of traits alleviating these physiological constraints to (i) optimize the optical density per biomass unit and, (ii) increase the resistance to photo-oxidation [5]. Decreasing overall absorption of photosynthetic active radiation (PAR) per cell [21] improves light distribution in PBR so that cells facing the surface absorb less photons, while those in inner layers become net contributors to carbon fixation [22]. Increasing resistance to photo-oxidative damage is expected to decrease photoinhibition. Indeed, preventing photoinhibition was reported to increase fitness [23] and provide carbon gain [24]. However, it is unclear whether manipulation of photoprotection traits might have beneficial effects for mass culture in PBR environment once weighted against the metabolic cost these processes have in algae. In this work, we report on the construction of *Chlorella vulgaris* strains combining enhanced light transmittance and resistance to oxidative stress by two steps of mutagenesis followed by phenotypic selection. The first selection round yielded the *pale-green PG*-*14* strain with increased photon use efficiency and higher biomass productivity in PBR. Further mutagenesis/selection allowed to select *pale-green* strains with increased tolerance to ^1^O_2_ (*SOR* strains). Selected *SOR* strains in PBR under strong irradiances showed further enhancement in productivity with respect to *PG*-*14*. Overall, these results show that resistance to oxidative stress is an important component of algal productivity. In addition, the traits responsible to improved optical properties can be combined with those providing oxidative stress resistance for the construction of domesticated algal strains with improved biomass yield for growth in PBRs.

## Results

### Isolation of PG-14, a pale-green mutant of *Chlorella vulgaris*

*Chlorella vulgaris* mutants that exhibited a pale-green (*PG*) phenotype with respect to WT were visually screened following EMS mutagenesis (Additional file 1: Figure S1A, B). Approximately 25,000 mutagenized lines were visual screened, and seven independent mutants were identified as putatively affected in Chl content per cell. All these mutants were capable of phototrophic growth and displayed different levels of depletion in Chl (Additional file 1: Figure S1C). Among the identified strains, p1–14 showed the highest growth rate in batch conditions (Additional file 1: Figure S2). It was, therefore, selected for further analysis and renamed as *PG*-*14*.

Pigment composition of both mutant and WT strains was determined after 5 days of growth, as shown in Table 1. *PG*-*14* showed a significant reduction of Chl content per cell (− 50%) when grown in minimal medium. The Chl *a/b* ratio was significantly higher in the mutant, with a value of 4.12 vs. 2.67 in WT, whereas the Chl/Car ratio was significantly lower in *PG*-*14* (2.25) with respect to the WT (4.03). These data suggest a depletion in the Chl *b*-rich antenna complexes (LHC) in *PG*-*14*.

**Table 1:** Pigment content, PSII maximum quantum yield (*F*_v_/*F*_m_) and PSII functional antenna size of WT and mutants *PG*-*14* and *SOR*

Genotype	Chl/cel l (pg)	Chl *a/b*	Chl/Car	*F*_v_/*F*_m_	PSII antenna size (${\text{T}}_{ 2/ 3}^{ - 1} 10^{ 3} {\text{ms}}^{ - 1}$)
WT	0.26 ± 0.03^a^	2.67 ± 0.22^a^	4.03 ± 0.22^a^	0.67 ± 0.03^a^	6.74 ± 0.49^a^
PG-14	0.13 ± 0.02^b^	4.12 ± 0.30^b^	2.25 ± 0.13^b^	0.69 ± 0.05^a^	4.40 ± 0.27^b^
SOR-1	0.13 ± 0.02^b^	4.12 ± 0.20^b^	2.24 ± 0.17^b^	0.67 ± 0.03^a^	4.17 ± 0.21^b^
SOR-5	0.11 ± 0.03^b^	3.96 ± 0.31^b^	2.24 ± 0.05^b^	0.66 ± 0.03^a^	4.37 ± 0.23^b^
SOR-6	0.13 ± 0.03^b^	4.20 ± 0.12^b^	2.25 ± 0.07^b^	0.66 ± 0.05^a^	4.29 ± 0.30^b^

Parameters were measured on dark-adapted cell suspension of WT, *PG*-*14* and *SOR* strains, upon 7 days of photoautotrophic growth in BG-11 medium in low light conditions (100 µmol photons m^−2^ s^−1^, 25 °C). Data are expressed as mean ± SD, *n* > 4. For each parameter measured, significantly different values among genotypes (ANOVA test, *p* < 0.05) are marked with different letters

### Stoichiometry of pigment-protein complexes and photosynthetic electron transport

**Fig. 1:**
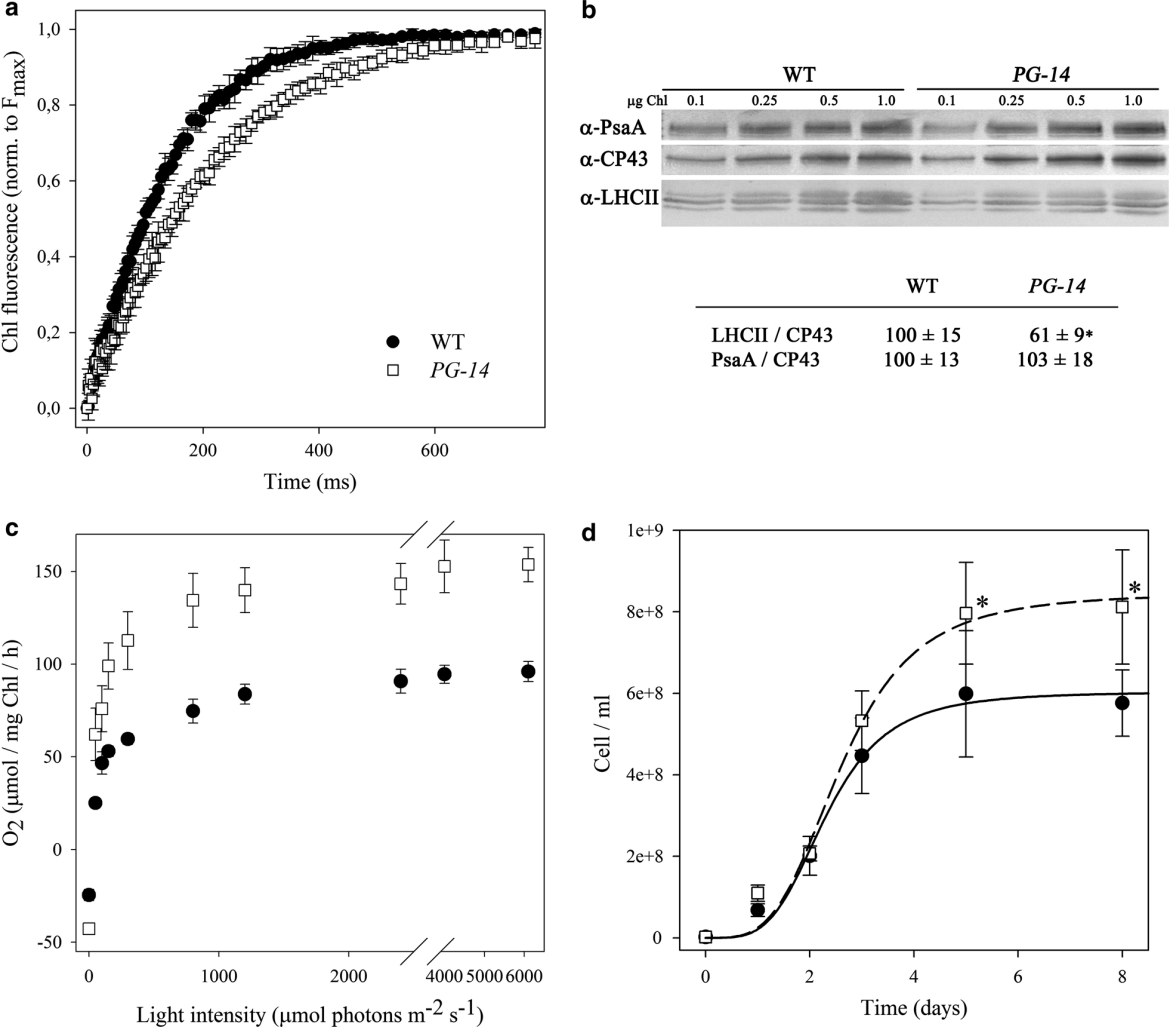
Characterization of *pale*-*green*-*14* (*PG*-*14*) mutant of *Chlorella vulgaris*. **a** PSII functional antenna size. Variable Chl fluorescence was induced with a green light (15 μmol photons m^−2^ s^−1^), on dark-adapted cells of WT and *PG*-*14*, in BG-11 medium supplemented with 50 μM DCMU. Data are expressed as mean ± SD, *n* = 10. The reciprocal of time corresponding to two-thirds of the fluorescence rise (*T*_2/3_) was taken as a measure of the PSII functional antenna size (see Table 1). **b** Immunoblotting used for the quantification of photosynthetic subunits. Immunotitration was performed with antibodies directed against individual gene products: LHCII, the major light harvesting complex of PSII; the PSII core subunit PsbC (CP43); the PSI core subunit (PsaA). The amount of Chls loaded for each lane is shown. Values significantly different (Student’s *t* test, *p* < 0.05) with respect to the WT are marked with asterisks. **c** Light-saturation curves of photosynthesis. Measured cultures (panels **a**–**c**) were grown in minimal BG-11 medium at 100 μmol photons m^−2^ s^−1^, in shaken flasks (120 rpm) illuminated from the top, photoperiod of 16/8 h light/dark, 25 °C. **d** Growth curves of wild type and *PG*-*14* mutant under autotrophic conditions. All experiments were performed in 1-L cylinders, illuminated with 1400 μmol photons m^−2^ s^−1^, 25 °C. Growths were performed in a semi-batch system fed with air/CO_2_ mix; the CO_2_ supply was modulated to keep the pH of the medium always below 7.1. Data are expressed as mean ± SD, *n* = 4

To determine whether the capacity of the antenna system to transfer absorbed energy to RCs was affected by the mutation, Chl fluorescence analysis was used to quantify the PSII operating efficiency. No significant differences were observed in *F*_v_/*F*_m_ between *PG*-*14* and WT (Table 1), suggesting that the maximal quantum yield of PSII photochemistry was not impaired in the *pg* mutant. Functional antenna size of PSII was measured on cell suspensions in the presence of DCMU, by estimating the rise time of Chl *a* fluorescence (Fig. 1a). The *T*_2/3_ of the Chl fluorescence rise is inversely related to the functional antenna size of PSII [25] and was reduced by ~ 35% in *PG*-*14* with respect to WT (Table 1).

These results were further confirmed through biochemical estimation of PSII antenna size. The level of selected thylakoid proteins was determined by immune-titration on thylakoids and expressed relative to WT once normalized to the PSII core complex (CP43) content (Fig. 1b). The LHCII abundance was reduced in the *PG*-*14* mutant to ~ 61% with respect to the WT level, while the PSI/PSII ratio was the same in both genotypes.

To investigate the functional properties of the photosynthetic machinery of the *PG*-*14* mutant, the light-saturation curve of photosynthesis was measured in photo-autotrophically grown cells (Fig. 1c). The rate of O_2_ release was shown to increase as a function of irradiance within the range of light intensities between 0 and 1000 µmol photons m^−2^ s^−1^. The increase was linear for both WT and *PG*-*14* at irradiances below 150 µmol photons m^−2^ s^−1^. The slope of the linear regressions of O_2_ yield vs. light intensity for WT and *PG*-*14* was 0.84 ± 0.21 and 1.32 ± 0.35, respectively, implying that the quantum yield of photosynthesis was significantly higher in the mutant with respect to WT. The light intensity for half-saturation of photosynthesis was similar in the two strains, at approximately 100 µmol photons m^−2^ s^−1^ (Table 2); irradiances higher than 1000 µmol photons m^−2^ s^−1^ were saturating for the O_2_ production in both WT and the *PG*-*14* mutant (Fig. 1c). In WT, the maximum rate of light-induced oxygen evolution *P*_max_ (photosynthesis net respiration) was reached at 2000 µmol photons m^−2^ s^−1^ and was equal to 96 ± 5 µmol O_2_ mg Chl^−1^ h^−1^. *PG*-*14* cells showed a significantly higher *P*_max_, i.e. (155 ± 11) (Table 2). Owing to the normalization of O_2_ production rate on Chl content, *P*_max_ is a measure of the Chl productivity for the two strains. The dark respiration rate was 25 ± 3 µmol O_2_ mg Chl^−1^ h^−1^ in the WT vs. 43 ± 3 in *PG*-*14* (Table 2), while it was the same in WT and mutant on a per cell basis. Altogether, these results imply that the *PG*-*14* mutant possesses an enhanced photosynthetic productivity with respect to the WT. To verify this hypothesis, growth rate and biomass productivity analysis were performed. Photoautotrophic growth was monitored over a period of 8 days in a lab-scale photobioreactor, a semi-batch cultivation system composed of 1-L glass cylinders exposed at a light intensity of 1400 µmol photons m^−2^ s^−1^. The system was fed with a flux of air and CO_2_, whose relative abundance was regulated by the pH of the medium in order to keep it within the range 6.8–7.2. The *PG*-*14* culture reached a cell concentration of ~ 8.1·10^8^ cell mL^−1^ at day five vs. 6.0·10^8^ cell mL^−1^ obtained in the WT (Fig. 1d), with a specific growth rate (µ) of the mutant which was significantly higher than the WT (2.00 day^−1^ for *PG*-*14* and 1.87 day^−1^ for WT, Table 3). Moreover, the mutant showed a higher mean biomass productivity, equal to 550 mg L^−1^ day^−1^, which was significantly higher (+28%) with respect to that in the corresponding WT (Table 3).

**Table 2:** Photosynthesis and respiration rates

Parameters	WT	PG-14	SOR-1	SOR-5	SOR-6
Half-saturation intensity (μmol photons m^−2^ s^−1^)	110 ± 24^a^	96 ± 41^a^	128 ± 6^a^	124 ± 9^a^	118 ± 29^a^
*P*_max_ (μmol O2 mg Chl^−1^ h^−1^)	96 ± 5^a^	155 ± 11^b^	146 ± 8^b^	143 ± 7^b^	150 ± 3^b^
Respiration (μmol O2 mg Chl^−1^ h^−1^)	25 ± 3^a^	43 ± 3^b^	40 ± 5^b^	47 ± 7^b^	42 ± 6^b^
Respiration (fmol oxygen cell^−1^ h^−1^)	6.4 ± 0.8^a^	5.5 ± 0.4^a^	5.2 ± 0.6^a^	5.6 ± 0.8^a^	5.4 ± 0.8^a^
*P*_max_/respiration (relative units)	3.9 ± 0.4^a^	3.6 ± 0.2^a^	3.7 ± 0.6^a^	3.1 ± 0.5^a^	3.6 ± 0.5^a^

Parameters were measured on dark-adapted cell suspension of WT, *PG*-*14* and *SOR* strains, upon 7 days of photoautotrophic growth in BG-11 medium in low light conditions (100 µmol photons m^−2^ s^−1^, 25 °C). O_2_ evolution/consumption was measured with a Clark-type oxygen electrode (Oxygraph, Hansatech). Data are expressed as mean ± SD (*n* > 4). For each parameter measured, significantly different values among genotypes (ANOVA test, *p* < 0.05) are marked with different letters

**Table 3:** Growth parameters of WT, *PG*-*14* and *SOR* strains, cultured in air/CO_2_ bubbling system

Genotype	Lab-scale, indoor PBR			
	1400 µmol photons m^−2^ s^−1^		50 µmol photons m^−2^ s^−1^	
	Mean increase of biomass (g l^−1^ day^−1^)	μ (day^−1^)	Mean increase of biomass (g l^−1^ day-1)	μ (day^−1^)
WT	0.43 ± 0.03^a^	1.87 ± 0.08^a^	0.059 ± 0.023^a^	0.94 ± 0.09^a^
PG-14	0.55 ± 0.02^b^	2.00 ± 0.07^b^	0.024 ± 0.004^b^	0.85 ± 0.13^a^
SOR-1	0.58 ± 0.03^b^	1.97 ± 0.04^a,b^	0.059 ± 0.008a	0.94 ± 0.08^a^
SOR-5	0.70 ± 0.03c	2.07 ± 0.03c	0.048 ± 0.002c	0.94 ± 0.09^a^
SOR-6	0.72 ± 0.04c	2.09 ± 0.03c	0.049 ± 0.015c	0.94 ± 0.08^a^

Biomass increase was measured by the determination of dry biomass accumulated after the cultivation period, divided by the number of days of cultivation (see Fig. 6). µ, specific growth rate, was calculated from the slope of logarithmic cell concentration curve. Growth was performed under autotrophic conditions, in 1-L cylinders, illuminated with either 1400 or 50 µmol photons m^−2^ s^−1^, 25 °C. Data are expressed as mean ± SD, *n* > 3. For each parameter and condition measured, significant different values among genotypes (ANOVA test, *p* < 0.05) are marked with different letters

### Isolation of pale-green, singlet oxygen-resistant strains

When experiencing excess light (EL) conditions, microalgae activate acclimatory responses involving induction of genes encoding products conferring enhanced tolerance to ^1^O_2_ stress [15].

**Fig. 2:**
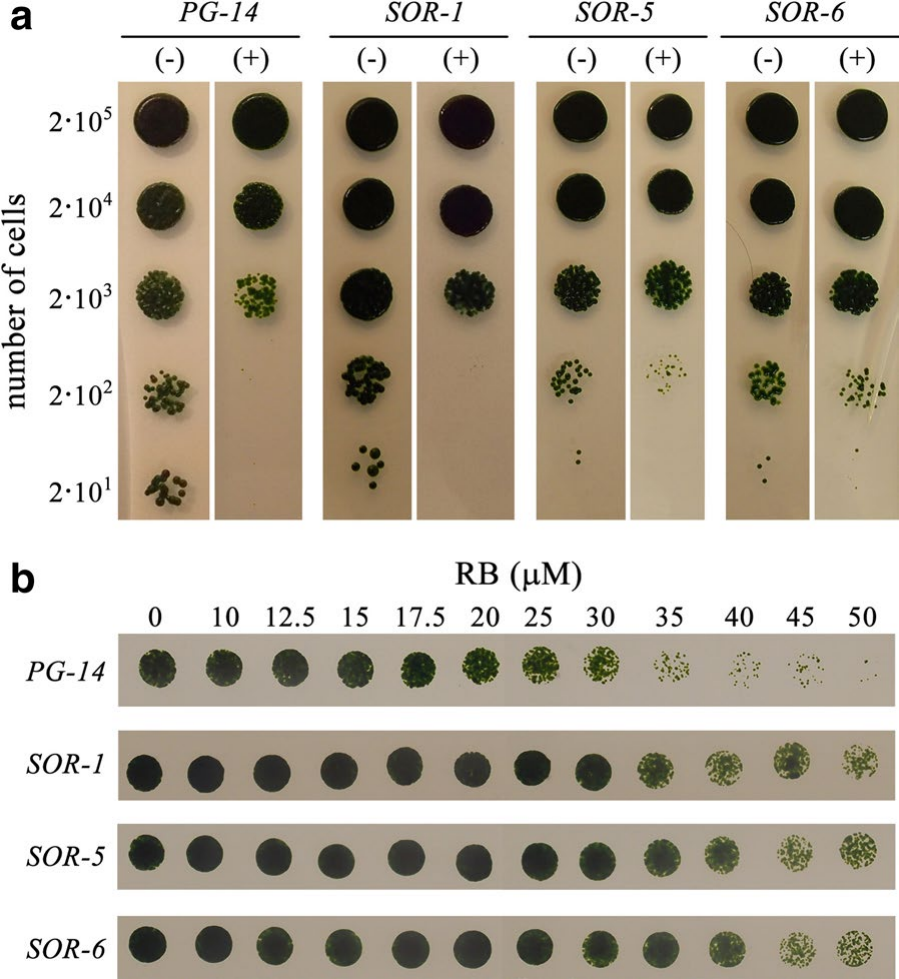
Isolation of singlet-oxygen-resistant (*SOR*) lines of *C. vulgaris*. **a** *SOR*-*1*, *SOR*-*5* and *SOR*-*6* strains were isolated as a ^1^O_2_-resistant mutant by growing on solid TAP medium either containing (+) or not (−) of Red Bengal (RB 12 μM). The amount of cells spotted is indicated in the left border. **b** Quantitative analyses of the resistance of *SOR* strains to oxidative stress. Cells were grown in liquid cultures with increasing concentration of RB (0–50 µM) for 24 h, then were spotted on TAP-agar for recovery. Plates were illuminated with 100 μmol photons m^−2^ s^−1^, photoperiod of 16-/8-h light/dark, 25 °C

To identify mutants with constitutively activated acclimation, we performed a screening for lines showing an increased tolerance to exogenous ^1^O_2_. The *PG*-*14* strain was mutagenized by EMS and plated onto agar containing the ^1^O_2_-photosensitizer Red Bengal (RB) used at the minimal concentration (12 µM) needed to inhibit growth of WT cells. Plates were exposed to a light–dark cycle (16:8 h) for 10 days, at which point 18 RB-resistant clones were isolated. To verify increased ^1^O_2_ resistance, colonies were picked to liquid medium in microtiter plates, and then re-evaluated for ^1^O_2_ tolerance by spotting cells in agar plates containing 12 µM RB (Fig. 2a). The three clones which passed the second screen were named as *singlet oxygen resistant* (*SOR*) mutants. No significant difference in the growth rates with respect to the parental line *PG*-*14* on control agar conditions were observed with these three mutant strains (Fig. 2a). RB significantly impaired growth of all genotypes; however, the effect was far more severe in the *PG*-*14* strains with respect to *SOR* mutants. The ^1^O_2_ resistance of the three *SOR* clones was quantified (Fig. 2b): cultures of *SOR*-*1*, -*5* and -*6* and the parental strain *PG*-*14* were exposed to increasing concentrations of RB (0–50 µM) in liquid cultures for 24 h, upon which, cells were spotted on agar plates. All *SOR* strains showed higher survival rates at high RB concentrations (≥ 25 µM) with respect to the parental strain.

**Fig. 3:**
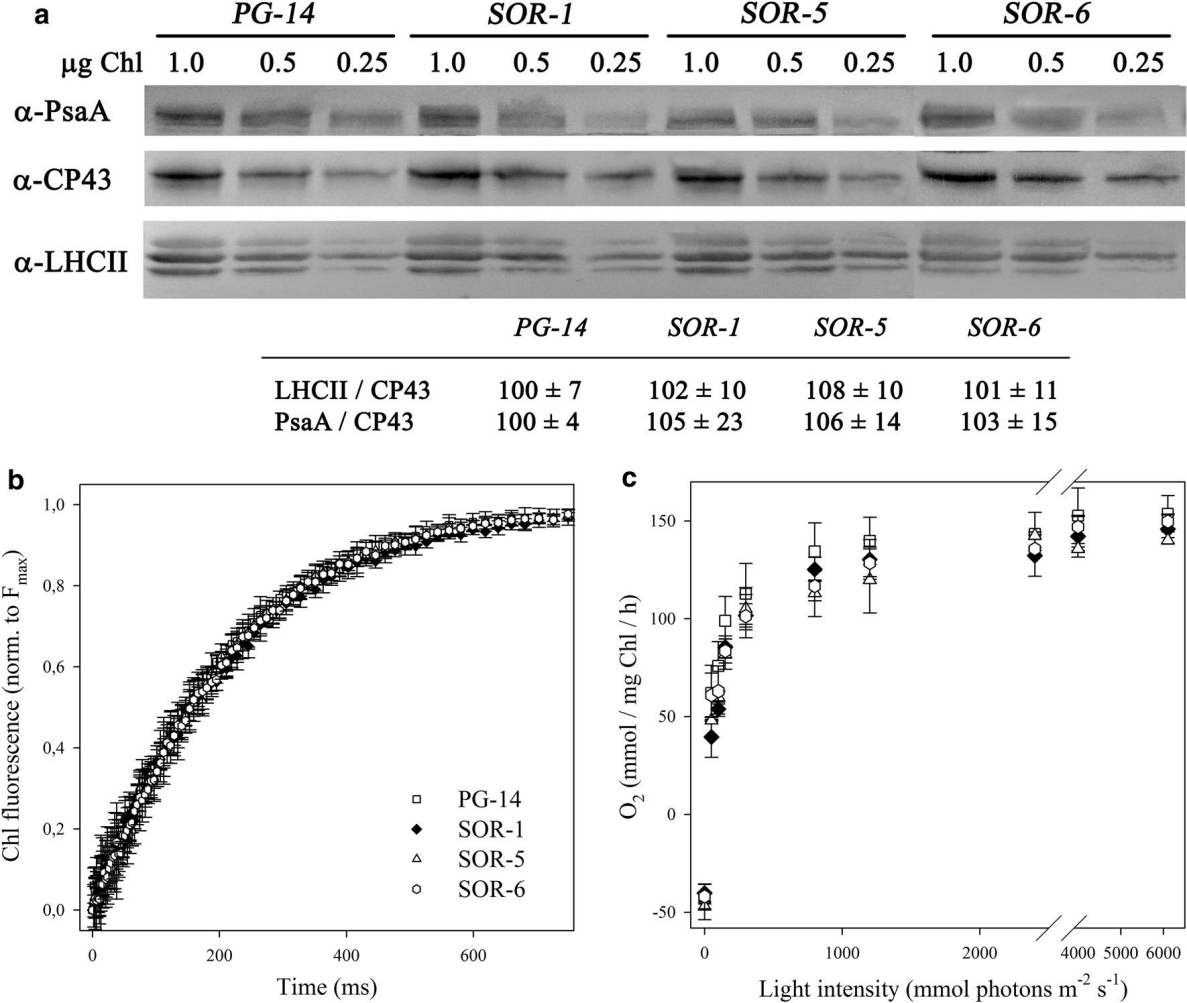
Photosynthetic characterization of *SOR* strains. **a** Immunoblotting titration of photosynthetic subunits in thylakoid membranes. PSII biochemical antenna size (LHCII/CP43 ratio) and PSI/PSII ratio (PsaA/CP43) are shown in the table. The amount of Chls loaded for each lane is shown. **b** PSII functional antenna size. Variable Chl fluorescence was induced on dark-adapted cells of *PG*-*14* and *SOR* mutant lines, in BG-11 medium supplemented with 50 μM DCMU. Data are expressed as mean ± SD, *n* = 10. See Table 1 for quantitative description of antenna size. **c** Light-saturation curves of photosynthesis. Data are expressed as mean ± SD, *n* = 4

*PG*-*14* and *SOR* mutants showed the same phenotype as for the Chl/cell, Chl *a/b* and Chl/Car ratios (Table 1), suggesting that the size of LHC antenna system was similar to the parental line *PG*-*14*. This was confirmed through immunotitration: both LHCII content and PSI/PSII ratio of *SOR* mutants were the same as measured in *PG*-*14* (Fig. 3a). No significant differences were observed in both PSII operating efficiency (*F*_v_/*F*_m_) and functional antenna size of PSII, with respect to the corresponding parental line (Fig. 3b, Table 1). In both *SOR* and *PG*-*14* strains, the light-saturation curves of photosynthesis (Fig. 3c) showed similar values for the maximum rate of light-induced oxygen evolution (*P*_max_), half-saturation intensity for photosynthesis and dark respiration rates on a per cell basis (Table 2), thus indicating that the *SOR* mutant strains maintained the reduction in antenna size and the enhanced photosynthetic productivity previously shown in *PG*-*14* cells.

### Sensitivity to photooxidative stress of SOR strains

Under strong light, microalgae undergo photooxidative stress [15]. Under such conditions, enhanced release of ^1^O_2_ leads to bleaching of pigments, lipid oxidation and a decrease of photosynthetic efficiency. Mechanisms evolved by photosynthetic organisms to limit photo-oxidative damage and acclimate to changes in the light environment include increasing Car pool size and a specific acclimation response enhancing ^1^O_2_-resistance. Therefore, the mutant strains *SOR*, with a constitutive up-regulation of protective mechanisms are expected to better perform under photooxidative stress conditions, by limiting pigment-protein damage and ultimately, photoinhibition.

**Fig. 4:**
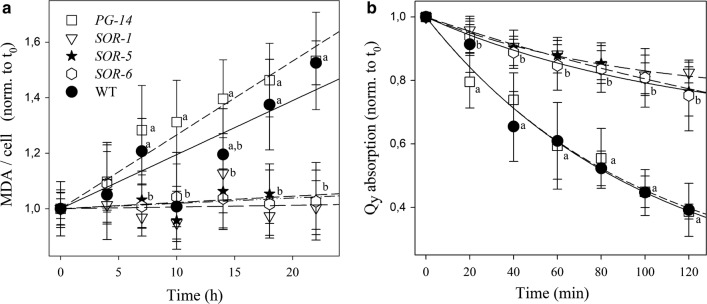
Photooxidation of *C. vulgaris* WT, *PG*-*14* and *SOR* mutant genotypes under photooxidative stress. **a** Cell suspensions were treated with 1400 µmol photons m^−2^ s^−1^ at 20 °C, and kinetics of malondialdehyde (MDA) formation were followed. MDA is an index of membrane lipid peroxidation, and was quantified by HPLC as thiobarbituric reactive substances. (B) Cell suspension of WT and mutant strains were treated with strong white light (14,000 µmol photons m^−2^ s^−1^, 20 °C) and the amount of Chl was evaluated by measuring the absorption area in the region 600–750 nm. See “Materials and methods” for details. Symbols and error bars show mean ± SD, *n* = 4. Values marked with the same letters are not significantly different from each other within the same time point (ANOVA, *p* < 0.05)

The sensitivity to EL stress of WT, *PG*-*14* and *SOR* mutant strains was assessed upon transfer of cells from control conditions to HL at 25 °C, then a time-course lipid peroxidation and pigment bleaching was measured (Fig. 4). When cells were exposed to 1400 μmol photons m^−2^ s^−1^ for 24 h, malondialdehyde production was 1.5 times higher in both WT and *PG*-*14* cells with respect to *SOR* (Fig. 4a) strains, implying a significantly lower level of lipid peroxidation in the latter.

We further examined liquid cultures of the WT and the mutants, at various times after transfer of low light-grown cells to extremely high irradiances (14,000 μmol photons m^−2^ s^−1^, at 25 °C). In both WT and *PG*-*14* cell suspensions, the Chl content decreased progressively upon exposure to HL until it reached ~ 40% of the initial value, after 2-h treatment (Fig. 4b). The rate of Chl bleaching was three times faster in WT or *PG*-*14* with respect to *SOR* strains (Fig. 4b).

To assess whether the *SOR* mutations affect the composition of the photosynthetic machinery, we determined the accumulation level of selected chloroplast proteins relative to the WT by immunotitration in EL-grown cells (Additional file 1: Figure S3). LHCII content was reduced in all mutants, ranging between 45 and 60% with respect to WT on a Chl basis. On the other hand, the PSI: PSII ratio was similar in all genotypes. Cytochrome *f* complex and ATP synthase (β subunits) were present in higher amounts in all mutants with respect to the WT. Rubisco was increased in all mutants (× 1.5–2 with respect to the WT level), with the only exception of *SOR*-*1* strain, whose Rubisco content accounted to 70% of the control strain (Additional file 1: Figure S3).

The antioxidant properties of the algal biomass are related to the efficiency of the detoxification mechanisms. To assess the antioxidant capacity of the extracts from WT and mutant lines, we resorted to more than one method, since the activity of an extract depends on its composition, polarity of extraction solvents and type of assay used.

**Fig. 5:**
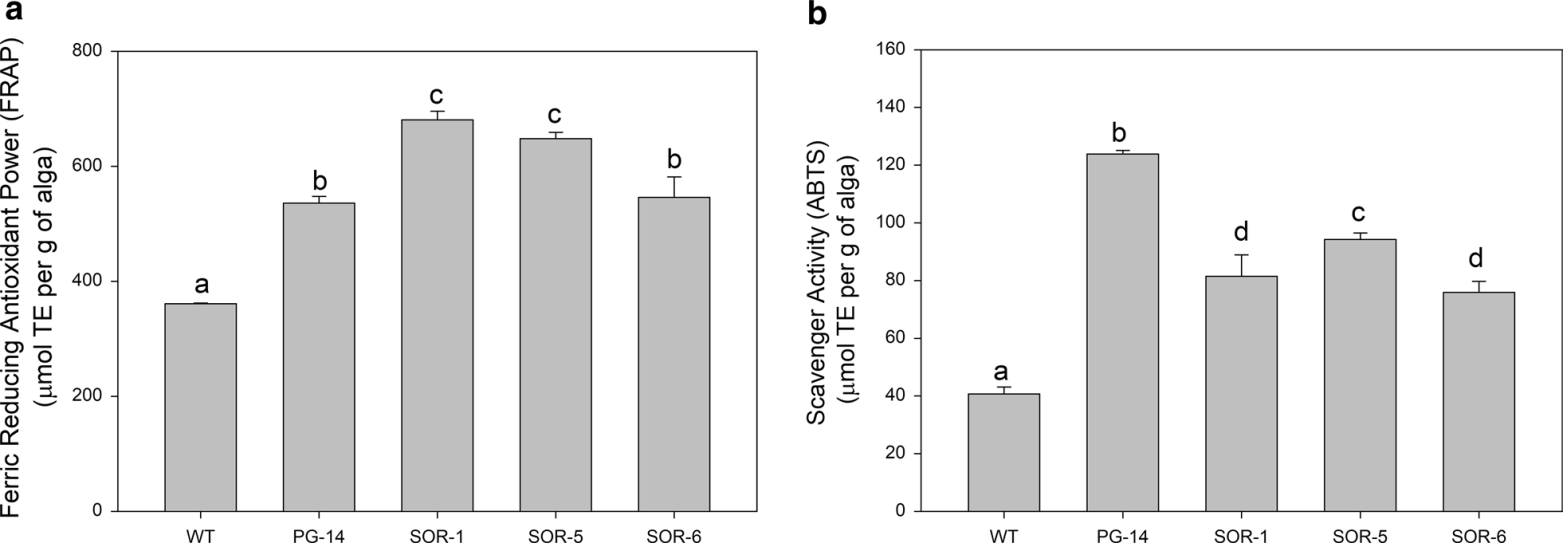
Comparison of antioxidant activity of whole-cell extracts from *C. vulgaris* WT and mutants *PG*-*14* and *SOR*. Antioxidant activity was measured by FRAP (**a**) and ABTS (**b**) assays. Within each panel, different letters indicate significant (ANOVA test, *p *< 0.05) differences. The values are the mean of 3 replicates, metric bars indicate SD

FRAP assay measures the capacity of an antioxidant in the reduction of the oxidant Fe^3+^ ion. When tested by FRAP, the levels of antioxidant activity of the different samples were as follows: *SOR* ≥ *PG*-*14 *> WT (Fig. 5a). ABTS (2,2′-azino-bis(3-ethylbenzothiazoline-6-sulphonic acid) assay, which requires a buffered aqueous solution, yielded very high scavenging activity in *PG*-*14* extracts; while all other mutants showed a significantly (*p* < 0.05) lower activity, even though far higher than the WT (Fig. 5b).

### Cultivation of WT and mutant strains in laboratory-scale photobioreactor

**Fig. 6:**
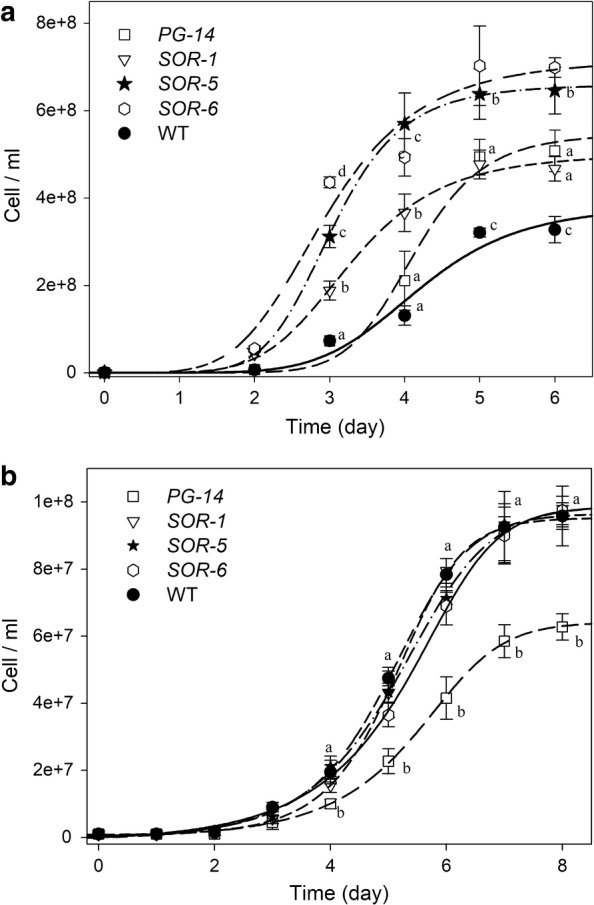
Growth curves of WT and mutant strains. Growth of WT, *PG*-*14* and *SOR* strains was performed under autotrophic conditions, at 25 °C, in 1-L cylinders, illuminated with either 1400 µmol photons m^−2^ s^−1^ (*panel* **a**) or 50 µmol photons m^−2^ s^−1^ (*panel* **b**). Cultures were maintained in a semi-batch system fed with air/CO_2_ mix; CO_2_ supply was modulated in order to keep the pH of the medium always below 7.2. Symbols and error bars show mean ± SD, *n* ≥ 6. Values marked with the same letters are not significantly different from each other within the same time point (ANOVA, *p* < 0.05)

The above results show that the mutant strains *SOR* have both enhanced efficiency of light energy conversion and higher tolerance to conditions of EL with respect to *PG*-*14* and WT strains, suggesting that they could have an enhanced light-to-biomass conversion yield. To verify such increased yield, growth rate and biomass productivity were further assessed in *SOR* lines. Photoautotrophic growth was monitored over a period of 6 days in the lab-scale 1L PBR at 1400 uE. Both the *SOR*-*5* and *SOR*-*6* cultures reached a cell concentration of about 6.8 10^8^ cell mL^−1^ at day six vs. 5.1 10^8^ cell mL^−1^ in the *PG*-*14* (Fig. 6a), with a spe cific growth rate (μ) higher than for *PG*-*14* (Table 3). Moreover, these mutants showed a higher mean biomass productivity, equal to 700 mg L^−1^ day^−1^, that was significantly improved (+30%) respect to the corresponding value for the *PG*-*14* (550 mg L^−1^ day^−1^) and for WT (430 mg L^−1^ day^−1^) (Table 3). The *SOR*-*1* mutant did display a faster growth rate (Fig. 6a); however, it did not show any significant enhancement in biomass productivity (580 mg L^−1^ day^−1^) with respect to *PG*-*14* (Table 3).

**Fig. 7:**
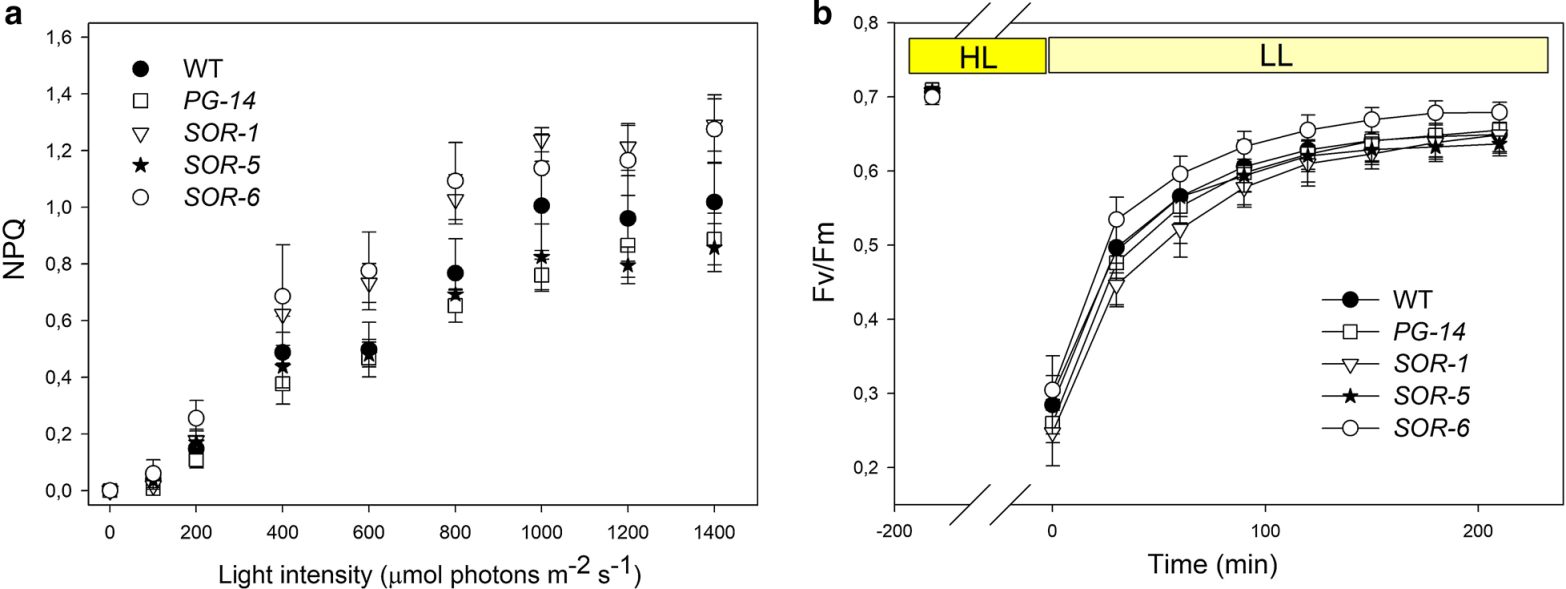
Analysis of room temperature chlorophyll fluorescence during photosynthesis under EL. **a** Chlorophyll fluorescence was monitored at 24 °C in dark-adapted cultures. Cell suspensions were illuminated for 20 min and the thermal energy dissipation (NPQ) was determined during steady-state photosynthesis. Symbols and error bars show mean ± SD (*n* = 4). **b** PSII repair efficiency was quantified on WT, *PG*-*14* and *SOR* strains plants by measuring *F*_v_/*F*_m_ (PSII photoinhibition) recovery in low light (LL—20 μmol photons m^−2^ s^−1^, 24 °C) after photoinhibitory treatment (HL—1800 μmol photons m^−2^ s^−1^, 24 °C, 3 h) that reduce the *F*_v_/*F*_m_ value to ~ 0,3 in all genotypes. Data are expressed as mean ± SD (*n *= 4)

Among the mechanisms possibly underlying the higher biomass yield of *SORs*, the most relevant is the Non-Photochemical Quenching (NPQ), which catalyzes dissipation, as heat, of the light energy absorbed in excess. In WT, *PG*-*14* and *SOR* genotypes, NPQ amplitude was measured at steady-state photosynthesis over a range of irradiances, showing differences mostly negligible (Fig. 7a). Moreover, recovery of *F*_v_/*F*_m_ upon photoinhibition (Fig. 7b) showed that all strains had the same behavior, implying that the higher growth of mutants was due to enhanced PSII repair.

The enhanced growth of the three *SOR* strain in HL is consistent with their truncated antenna system and resistance to ROS. However, it is unclear whether it is also due to enhanced efficiency in light-use efficiency. To explore this possibility, we grew these strains as well as WT and *PG*-*14* in limiting-light conditions (Fig. 6b). At 50 µmol photons m^−2^ s^−1^, *PG*-*14* had a lower grow rate with respect to WT as expected from its reduced antenna size. The three *SOR* mutants had a growth kinetic and final biomass yield similar to WT, despite they had a reduced antenna size, similar to *PG*-*14* (Fig. 6b, Table 3). These results are consistent with enhanced growth rate of *SOR* mutants being caused not merely by enhanced ROS resistance plus enhanced growth penetration in the culture, but also by enhanced light-use efficiency in low light.

### Investigation of lipid production as a response to nitrogen starvation

Lipid yield is a key parameter to be considered when aiming to produce biofuels. Stress factors, such as excess light [26, 27] and nitrogen starvation [28, 29], trigger lipid accumulation in algae. ROS likely participate as molecular mediators of stresses in algae as suggested by the correlation between lipid content and intracellular ROS level in *C. vulgaris* [30]. Because of this relation, the higher resistance to oxidative stress observed might prevent oil productivity by impairing ROS cross-talk signals. We, therefore, evaluated the lipid productivity in our selected mutants, under a two-stage cultivation protocol, in which microalgae were first grown in the standard BG-11 medium to achieve high cell density and then transferred to a modified BG-11 medium with limiting N source. At the end of growth phase, oil content per DW was determined gravimetrically. Dry biomass from WT contained ~ 25% oil, in accordance with previous quantification in the same species [12]; N-deprivation brought about oil content of *PG*-*14* and *SORs* to the same level than WT cells (Table 4). The fatty acid content and composition of oil fraction were also determined: dry biomass from *PG*-*14* and *SOR* mutants were enriched in fatty acids than the WT as follows: *PG*-*14 *> *SOR5/6 *> *B1*; EL-grown WT strain showed high relative amounts of C16:0 fatty acids (~ 18% of total acyl chains) and high proportions of mono-unsaturated C18:1 (~ 28%) and di-unsaturated C16:2 and C18:2 fatty acids (~ 44%), which overall accounts for more than 90% of total acyl chains in *C. vulgaris*. Comparison of the fatty acid profiles of the mutant strains revealed that the C16:2 content was reduced in all mutants (− 15/− 35% vs. WT); while the C16:0 content was essentially unaffected. *PG*-*14* and *SOR* mutants underwent changes in the C18 composition, with a significant increase of C18:1 in *PG*-*14* and *SOR* mutants vs. WT and a corresponding decrease of C18:2 acyl chain (Additional file 1: Table S1).

**Table 4:** Lipid content of algal biomass

Genotype	Total oil content (% DW)
WT	25.1 ± 2.7^a^
PG-14	23.2 ± 0.8^a^
SOR-1	22.2 ± 2.3^a^
SOR-5	21.9 ± 2.2^a^
SOR-6	27.5 ± 2.6^a^

Total lipid content was determined gravimetrically on the dry biomass, from WT and mutant cultures grown for 7 days in standard, nutrient-rich BG-11 medium at 1400 µmol photons m^−2^ s^−1^ 25 °C, and then moved for further 4 days of growth in modified BG-11 medium with limiting N source. Data are expressed as mean ± SD, *n* = 4. Significant different values in oil content among genotypes (ANOVA test, *p* < 0.05) are marked with different letters

## Discussion

### Reduction of antenna size improves light-use efficiency

Limiting factors for large-scale algal biomass production include the inefficient use of photons under mass culture conditions, due to high optical density of the cell suspension and the generation of steep light gradients. Biomass productivity can be improved by engineering optical properties of strains. Previous work targeted genes, such as *TLA1* [31, 32], which control antenna size in the model species *C. reinhardtii*, yielding enhanced productivity. A forward-genetic approach was adopted in species with a high market interest, such as *C. sorokiniana* and *N. gaditana*, involving random mutagenesis and screening for desired traits. Truncated antenna mutants were selected for both species, and exhibited increased photon use efficiency and biomass yield in dense cell suspensions, which are typical of industrial PBRs [22, 33]. In this work, we used a similar approach with *Chlorella vulgaris*, a robust, interesting species for industrial applications. In a first screen, we searched for pale-green phenotype to establish a background strain for further domestication by incorporating the low optical density trait [21, 22, 32], thus increasing light penetration and light-to-biomass yield.

Among seven pale-green mutants recovered from screening 25,000 colonies, a range of reduction in Chl content between 25 and 60% with respect to WT strain was obtained (Additional file 1: Figure S1C). Clearly, reduction of Chl content per se was not sufficient for improving light-use efficiency in all strains (Additional file 1: Figure S2). In fact, four mutants (*p1*–*14*, *p1*–*43*, *p1*–*47* and *p2*–*77*) showed a productivity higher than WT, while the others performed similar to the control genotype despite their *pg* phenotype. Strains *p2*–*25* and *p2*–*36* had a similar Chl content per cell as *PG*-*14* and yet performed differently. This is consistent with the previous reports with two low Chl/cell mutants in *Cyclotella* sp. [34] whose productivity respect to WT was not improved in PBRs. Indeed, random mutagenesis may well affect multiple genes, thus influencing cell metabolism and impairing growth. Therefore, a full photosynthetic characterization of mutants obtained by chemical/UV mutagenesis is needed to ensure that pale-green mutants are not affected in their photosynthetic performance other than by reducing their LHCII content [22, 35]. We chose *PG*-*14* based on its defect in PSII antenna size, consisting in a LHCII content of 61% with respect to WT (Fig. 1b). Fluorescence induction in cells infiltrated with DCMU confirmed that *PG*-*14* had a marked reduction of the PSII functional antenna size as compared to WT (Fig. 1a). In contrast, the PSII: PSI ratio was unaffected with respect to WT level (Fig. 1b).

Photosynthetic yield was significantly enhanced in *PG*-*14* vs. WT. Indeed, the *P*_max_ of *PG*-*14* was 60% larger than in WT at saturating irradiances (Table 2), implying that the undercut in antenna size did not negatively impact on photosynthesis. These results are consistent with those reported for *C. reinhardtii* mutants *tla1* [35] and *tla3* [21].

Owing to the double light-harvesting and photoprotective function of LHC proteins, it is relevant to consider whether mutations increase susceptibility to photoinhibition [19] as observed in the *ch1* mutation [36], by preventing LHC assembly, which leads to overproduction of ^1^O_2_ and enhanced photooxidation [37]. The light-saturation curve of photosynthesis showed no decline of O_2_ evolution even at very high light intensity (6000 μmol photons m^−2^ s^−1^, Fig. 1c), whereas the lipid peroxidation and rate of Chl photobleaching were the same in *PG*-*14* and WT cultures exposed to high light (Fig. 4). We conclude that 50% loss of LHC per PSII does not significantly affect photo-tolerance of *C. vulgaris* cells under the tested growth conditions. Finally, cell growth rates and biomass yield were measured in the long-term cultivation of dense algal suspensions under very high irradiance (1400 μmol photons m^−2^ s^−1^). During 8 days of growth, *PG*-*14* showed a significant increase of productivity with respect to WT, both as biomass increment per day and maximal level of biomass reached at the end of the growth period (Fig. 1d, Table 3). In conclusion, characterization of *C. vulgaris PG*-*14* strain confirmed that selection for reduced optical cell density is a viable strategy to obtain higher productivity.

### Enhanced resistance to oxidative stress differently affects the growth rate

How do the *SOR* mutations contribute to the increased growth rate relative to *PG*-*14*? Photosynthetic organisms have evolved a number of photoprotective mechanisms to limit photooxidative damage [15], the latter being an unavoidable consequence of the presence of highly reactive intermediates during oxygenic photosynthesis. These mechanisms are active in (i) preventing over-excitation of reaction centers by quenching either ^1^Chl* [16] or ^3^Chl* states [37], thus avoiding ROS formation, or (ii) detoxifying ROS [38]. The first class includes the activation of energy dissipation into heat thus relieving the excitation pressure on PSII [16], and long-term physiological acclimation processes, some of which induce the re-organization of the photosynthetic apparatus [15] upon triggering by signal pathways activated by oxidative products [39]. The antioxidant defence mechanisms include enzymatic- (APX, SOD, CAT, etc.) and non-enzymatic antioxidants (carotenoids, tocopherols, ascorbate, glutathione) [40]; in particular, the thylakoid-bound antioxidants carotenes and xanthophylls play an irreplaceable role as structural components of the photosynthetic apparatus and photoprotective molecules, crucial in both quenching ^1^O_2_ and inhibiting lipid peroxidation [41].

Among the first class of mechanisms, possibly underlying the higher biomass yield of *SORs*, NPQ is the most relevant. NPQ catalyzes dissipation, in form of heat, of the light energy absorbed in excess. In microalgae, the mechanism is activate by LhcSR (light-harvesting complex stress-related) protein [42]. In WT, *PG*-*14* and *SOR* genotypes, NPQ amplitude was measured at steady-state photosynthesis over a range of irradiances, showing differences mostly negligible (Fig. 7a), consistent with the similar LhcSR content in all the strains (Additional file 1: Figure S3). Thus, we conclude that the differences in growth of *SOR* lines were not due to enhanced NPQ.

The antioxidant activity of *PG*-*14* and *SOR* mutants was in general higher than the WT, irrespective of the antioxidant assay used. *SOR*-*1* showed the highest antioxidant power when tested with FRAP, a reagent that evaluates the antioxidant activity by reducing a metallic ion (Fig. 5a). This assay has been used to evaluate both lipophilic substances such as tocopherol as well as more polar antioxidants as ascorbic acid [43]. On the other hand, *PG*-*14* showed the highest antioxidant activity with the ABTS assay (Fig. 5b). The latter assay reacts with both phenolic compounds and carotenoids [44] and correlates with the higher content of carotenoids of this mutant with respect to WT (Table 1). However, since the carotenoid level of all mutants is not significantly different (Table 1), other compounds (e.g., phenolics or other natural antioxidants) might be more abundant in *PG*-*14* cells than *SOR* mutants. Lower MDA accumulation and higher resistance to photobleaching in EL (Fig. 5) suggest either a reduced ^1^O_2_ release of *SOR* vs. *PG*-*14* and WT, or a more effective scavenging activity of ^1^O_2_ in the chloroplasts, being this ROS unstable and suggested as unable to leave the plastid compartment [45]. Reduced ^1^O_2_ release within the chloroplasts appears unlikely, being content of the major photosynthetic components identical in *PG*-*14* and *SOR* strains (Additional file 1: Figure S3). Regarding the antioxidant capacity, it is worth noting that *SOR*-*6* cells, namely the fastest-growing strain, did not show higher antioxidant ability than the control genotype *PG*-*14*, with both methods used; the other two *SOR* strains showed a slightly higher antioxidant capacity when probed by FRAP methods, while lower capacity than *PG*-*14* when probed by ABTS. Similar results were obtained with other two antioxidant assays, namely DPPH and Folin–Ciocalteau (data not shown). In an attempt to reconcile such contrasting results, it can be hypothesized the antioxidant assays here chosen is more sensitive to specific ROS, thus might have underestimated the contribution of ^1^O_2_-specific detoxification mechanisms, although more active in *SOR* mutants. Alternatively, the scavenging potential of *SOR* cells might not be the main factor improving photosynthetic performance in EL.

The higher resistance of *SOR* strains to photooxidative stress was not due to enhanced capacity for PSII repair process: indeed, the recovery of *F*_v_/*F*_m_ (quantum yield of PSII) upon photoinhibitory treatment showed that all genotypes showed a similar behavior (Fig. 7b).

Interestingly, these mutants showed a biomass productivity significantly higher with respect to the corresponding value for the *PG*-*14* even when grown in limiting-light conditions (50 µmol photons m^−2^ s^−1^), showing biomass productivities similar to WT culture (Fig. 6b, Table 3). Similar results were obtained in growth assay carried out at lower irradiance (20 µmol photons m^−2^ s^−1^, data not shown). These results are unexpected in mutants with truncated light-harvesting antenna size, in which photosynthetic efficiency decreases at sub-saturating irradiances due to limitation in PSII cross section.

Indeed, enhanced growth in low light despite truncation in antenna size suggests that the enhanced light-use efficiency is obtained in *SOR* genotypes, and contributes to the enhanced biomass production. Clearly, this is not due to enhanced PSII repair not to decreased NPQ with respect to WT and *PG*-*14,* and its nature is unclear based on present results. Hypothesis includes altered thylakoid architecture, change in composition or relative abundance of LHC proteins, altered kinetic in the remodeling of photosynthetic membranes in response to illumination [46] or up-regulation of plastidial regulatory elements [47, 48]. The elucidation of this mechanism will be the object of further analysis including identification of mutations and physiological analysis of these genes, to identify genetic elements potentially useful for enhancing primary productivity.

Alternatively, higher biomass yield in EL might not be exclusively due to either improved antioxidant networks or enhanced light-use efficiency in *SOR* strains. Pal et al. [49] showed that activation of stress response upon ^1^O_2_ induction was effective in increasing resistance to oxidative stress in *C. reinhardtii* without altering antioxidant levels. Indeed, depending on its concentration, ^1^O_2_ may either cause oxidative damage or act as “second messenger” in cell signal transduction. In algae, similar to reports on plants [50], PSII is the cellular major source of ^1^O_2_. However, due to its high reactivity and short lifetime, ^1^O_2_ is not considered as directly involved in chloroplast-to-nucleus signaling; rather, plant cells perceive ^1^O_2_ as signal which controls a number of stress-response mechanisms [51]: in *Arabidopsis*, EXECUTER proteins (EX1 and EX2) are involved in the regulation of the ^1^O_2_-mediated genetic response [52, 53]; in *Chlamydomonas*, specific responses to ^1^O_2_ vs. other ROS was attributed to specific promoter regions [54, 55]. Moreover, a moderated ^1^O_2_ release under EL stress affects susceptibility of *C. reinhardtii* cells to subsequent, more severe stresses [49]. In plants, induction of stress acclimation allowed higher protection against PSII photoinhibition upon harsher conditions: acclimation activates a subset of ^1^O_2_-responsive genes in WT plants, while represses them in *ex1/ex2* plants. However, the extent of photosensitivity in WT and *ex1/ex2* plants was the same upon acclimation [53], thus suggesting that it relies on extensive cross-talk with different stress-related signaling pathways.

A set of plastid-derived signals were found to be involved in the adaptation of cell physiology to the changing environmental conditions: these include tetrapyrroles, metabolites such as 3-phosphoadenosine-5-P (PAP) and methylerythritol cyclo-PP, ROS and cleavage products of carotenes and/or phytofluene, as well the redox state of stroma and the organellar gene expression (reviewed in [56, 57]. Under photooxidative stress, oxidized derivatives of β-carotene such as β-cyclocitral (β-CC), β-ionone and dihydroactinidiolide (dhA) act as signaling molecules, inducing transcriptomic responses associated with enhanced resistance to photoxidative stress [39, 58, 59]. Release of carotenoid oxidation products was reported not only in plants but also in cyanobacteria [60], while the small zinc finger proteins MBS were shown to mediate the ^1^O_2_-dependent transcriptomic response in both *Chlamydomonas* and plants, thus suggesting these sensing mechanisms are ubiquitous in the green lineage.

The task of identifying the signaling pathway affected in *SOR* mutants is complex due to integration of multiple signaling cascades whose components are still poorly understood and involving considerable cross-talk aimed at sustaining cell homeostasis during stress responses as shown by interaction between elements of PAP and β-CC retrograde signaling. Such interactions promote gene reprogramming and enhance tolerance to photoinhibition as shown by the positive effect of pre-treatment with either PAP or β-CC before stress treatment [58, 61].

We speculate that higher phototolerance in *SOR* mutants might be related to a constitutive activation of one or more of the above-mentioned signal transduction pathways. High-productivity phenotype of SORs suggests that multiple defense processes might be affected, possibly due to mutations in global regulators of photoprotection response in algal cell. Future identification of these mutations will possibly allow pinpointing new components of ^1^O_2_-mediated signaling control and their role in different cellular protection mechanisms.

### Influence of ROS-resistance traits on stress-induced lipid production

While excessive ROS release causes irreversible damage to cellular structures, ROS formed under mild photooxidative stress conditions promote lipid accumulation in oleaginous microorganisms [62], possibly by a complex signaling pathway that triggers up-regulation of the enzyme ACCase and increases C flux into the pathway of fatty acid biosynthesis [63]. Indeed, [30] found that intracellular lipid content in *C. vulgaris* is correlated with hydroxyl radical levels. Moreover, oxidative stress perception by the endoplasmic reticulum promotes lipid droplets formation [64]. Overall, direct experimental evidences of an association between ROS and intracellular lipids are still scarce, and details on the molecular mechanisms of ROS-mediated lipid accumulation are missing. However, if ROS are obligate mediators of lipid accumulation by complex signaling pathways, then we might expect an altered lipid yield in an individual mutant possibly affected in such stress-response pathways. Quantification of oil content, upon short-term nitrogen-starvation treatment of cultures, ruled out the hypothesis: indeed, oil content was essentially the same in all strains analyzed, while fatty acid content of dry biomass was significantly higher in all mutants than WT. We only measured a decrease of C16:2 acyl chain fraction in mutant strains vs. WT; a significant increase of C18:1 acyl chain in mutants vs. WT and a corresponding decrease of C18:2 acyl chain, the most abundant PUFA of *C. vulgaris,* was also shown. These results contrast with former evidence that higher ROS release obstructs PUFA biosynthesis [65]: indeed, kinetic of MDA formation in EL suggests PUFA peroxidation is lower in *SOR* vs. WT and *PG*-*14* strains. Rather, such a change in lipid composition points again to an altered signaling cues in *SORs* with respect to control genotypes.

## Conclusions

Our results show that domestication of WT strains, by both modulating antenna size to improve light penetration and enhancing resistance to excess light, is an effective strategy in the development of microalgal strains optimized for mass culture and these effects are additive towards a higher biomass productivity and stress resistance. Further work will be undertaken for the identification of genes affected in the SOR mutants by DNA and RNA sequencing. Owing to the impossibility of carrying on genetic crossing in *Chlorella*, pyramiding positive traits will need reliable transformation and gene editing procedures that are still to be implemented in this genus. Moreover, we have shown a specific effect for *SOR* mutations in improving light-use efficiency. Indeed, besides the enhanced ^1^O_2_ scavenging effect of *SOR* genotypes, an additional, still unknown, mechanism is needed to explain their higher growth rates in limiting-light conditions. Identification of this mechanism will be the subject of future research.

## Materials and methods

### Strains and culture conditions

*Chlorella vulgaris* WT strain was obtained from the SAG Culture Collection of Algae (Goettingen University, Germany, http://www.uni-goettingen.de/en/catalogue-of-strains/185049.html) as SAG strain number 211-11p. Cells were maintained on TAP-agar plates [66] and grown in either minimal (BG-11) [67] or rich (TAP) media. Shaken flasks (120 rpm) were illuminated from the top with 100 μmol photons m^−2^ s^−1^, photoperiod of 16/8 h light/dark, 25 °C; irradiance was provided by warm-white LEDs (Epistar 35mil Chip High Power LED, warm white LEDE-P20B-DW, Wayjun Tech., Shenzhen, China). For all physiological and biochemical measurements, cultures were harvested during the logarithmic growth phase (~ 1·10^8^ cells mL^−1^). For short-term nitrogen-starvation experiments, cells were grown for 4 days in standard BG-11 medium containing excess nitrogen source (NaNO_3_ 1.5 g L^−1^) at an irradiance of 1400 µmol photons m^−2^ s^−1^, 25 °C, with a photoperiod of 16/8 h light/dark; cells were then collected by centrifugation, washed twice with sterile water, and re-suspended at 1·10^8^ cells mL^−1^ in a modified BG-11 medium with limiting N source (NaNO_3_ 0.07 g L^−1^) and further grown for 3 days.

### Mutagenesis and screening protocols

*C. vulgaris* WT cells in liquid cultures were harvested by centrifugation at the exponential phase of growth (~ 1·10^8^ cells mL^−1^), re-suspended in fresh TAP medium to 5·10^7^ cells mL^−1^, and treated with ethyl methanesulfonate (EMS). The survival curve for mutagenesis with EMS was carried out to determine the mutagen concentration which resulted in around 5% of cells viability. Upon 2-h maintenance in the dark, to prevent light-activated DNA repair, cells were plated at 100-fold dilution on TAP-agar medium and exposed to 100 µmol photons m^−2^ s^−1^. Single colonies appeared after 14 days. The ones showing a pale-green phenotype were identified by direct sight inspection, inoculated onto fresh minimal medium, grown in the light for seven days, and the Chl content per cell was determined. This procedure allowed to isolate the pale-green mutant strain *PG*-*14*. Chemical mutagenesis was repeated on the strain *PG*-*14* to isolate singlet oxygen-resistant mutant strains. The photosensitizer Red Bengal (RB) was used as selection method: RB is a chemical producing ^1^O_2_ when exposed to white light [68]; thus, the screening of the mutants was performed by their resistance to the exogenous ^1^O_2_. A wide range of concentrations of chemical were tested to find out the minimal concentration which inhibited grown of the PG-14 strain. SOR (singlet oxygen resistant) mutants were selected by plating mutagenized *PG*-*14* cells on TAP-agar plates containing 12 μM RB, which were then exposed to 100 μmol photons m^−2^ s^−1^ to initiate the selection for resistant clones. ^1^O_2_-resistance of selected mutants was tested by serial dilution of a concentrated culture (~ 5·10^7^ cells mL^−1^), spotted onto plates containing 12 μM RB and maintained in the light for several days. Estimation of resistance to exogenously generated ^1^O_2_ was done by transferring aliquots of culture (5·10^7^ cells mL^−1^) into a multi-well plate and adding RB (0–50 µM). Cells were grown for 24 h at 100 μmol photons m^−2^ s^−1^, then 10 μL from each well were spotted on TAP-agar plates.

### Cell count and pigment analysis

Cell density was measured using an improved Neubauer hemocytometer. Pigments were extracted from intact cells with 100% dimethyl-formamide. The supernatant of each sample was recovered after centrifugation (10 min at 15,000*g*, 4 °C), diluted in acetone and pigments were separated and quantified by HPLC [69].

### Gel Electrophoresis and Immunoblotting

For SDS-PAGE and immunotitration analysis, cells were resuspended in Loading Buffer (5% glycerol, 1% SDS, 2.5% 2-mercaptoethanol, 0.1 M Tris, 0.1 M Tricine pH 8.45) and grinded in a tissue homogenizer (Precellys, Bertin, France) by adding a ceramic lysing matrix. The supernatant of each sample was recovered after centrifugation (10 min at 15,000*g*, 4 °C) and Chl content of extracts was determined. SDS-PAGE analysis was performed with the Tris-Tricine buffer system [70]. For immunotitration [71], a range of total protein extract corresponding to 0.1–2.0 µg of Chl were loaded for each sample and electroblotted on nitrocellulose membranes. Proteins were detected with primary antibodies (home-made: α-CP43, α-Rubisco, α-LhcSR; from Agrisera: α-PsaA AS06-172-100, α-Cyt*f* AS06-119, α-ATPase β subunit AS05-085) and an alkaline phosphatase-conjugated secondary antibody (Sigma-Aldrich A3687). Signal amplitude was quantified using the GelPro 3.2 software (Bio-Rad).

### Measurements of photosynthetic activity

The oxygen evolution activity of the cultures was measured at 25 °C with a Clark-type O_2_ electrode (Hansatech, UK) upon illumination with white light provided by a halogen lamp (Schott, Germany). Samples of 2 mL cell suspension (~ 5·10^7^ cell mL^−1^) were loaded into the oxygen electrode chamber; 3 mM NaHCO_3_ was added to the cell suspension prior to the O_2_ evolution measurements to ensure electron transport was not limited by the carbon supply.

### In vivo chlorophyll fluorescence analysis

Fluorescence induction kinetics was recorded with a home-built apparatus as previously described [72]. Variable fluorescence was induced with a green light of 7 μmol photons m^−2^ s^−1^ at RT, on cells suspensions (~ 5·10^7^ cells mL^−1^) in BG-11 medium containing 100 µM DCMU. The reciprocal of time corresponding to two-thirds of the fluorescence rise (*T*_2/3_) was taken as a measure of the PSII functional antenna size [25]. Quantum efficiency of PSII (*F*_v_/*F*_m_) was measured on cell suspension, dark-adapted for 20 min, with a PAM 101 fluorimeter (Heinz-Walz, Germany). The light dependence of NPQ during photosynthesis was measured through Chl fluorescence on dark-adapted cell suspension at RT with a Fluor-Cam 700MF (Photon Systems Instruments, Brno, Czech Republic); NPQ was calculated according to [73] at steady-state photosynthesis (upon 20 min illumination).

### Determination of the sensitivity to photooxidative stress

The extent of lipid peroxidation in cells was estimated by measuring malondialdehyde (MDA) formation, as an indirect quantification of lipid peroxides [74]. Quantitative evaluation was done by transferring 2 mL aliquots of WT and mutant cell suspensions (~ 5·10^7^ cell mL^−1^, in BG-11) into a 24-well culture plate, kept on a rotary shaker and illuminated for 2 days with high light (1400 µmol photons m^−2^ s^−1^, 25 °C). Samples (2·10^5^ cells) were taken for analysis during a period of 48 h, and frozen in liquid nitrogen. MDA content of aliquots was quantified as previously described [75].

The photobleaching kinetics of Chl cell content were measured on cell suspensions (~ 5·10^7^ cell mL^−1^, in BG-11 + 0.03% w/v agarose) using actinic light intensities of 14,000 μmol of photons m^−2^ s^−1^ for 2 h; temperature of samples was maintained at 25 °C. During the illumination, the absorbance area between 600 and 750 was recorded; the initial and maximal absorbance were set, so the same absorbance area was used in the wavelength range 600 nm < *λ* < 750 nm for all the samples.

### Growth analysis

Growth experiments were performed at 25 °C in a home-built photobioreactors, composed of glass cylinders with a maximum light path of 8 cm and a working volume of 1 liter each [22]. Cultures were continuously mixed with a flux of air and CO_2_. The ratio of compressed air and CO_2_ was automatically adjusted to keep the pH of the medium within the range 6.8–7.2. Each autotrophic batch cultivation was carried out in duplicate. Illumination was provided by a panel of warm-white LEDs (Epistar 35mil Chip High Power LED, warm white LEDE-P20B-DW), microalgae were exposed to an irradiance of 1400 µmol photons m^−2^ s^−1^, with a photoperiod of 16/8 h light/dark. The parameters determined to monitor cell growth were cell number and dry biomass weight, for which the washed cell pellets were dried overnight in a lyophilizer. The inoculum size was 1·10^6^ cell mL^−1^.

### Determination of total lipid content and lipid composition

Total lipids were extracted from 100 mg lyophilized biomass from 3 days nitrogen-starved cultures, homogenized by 4 cycles of 30 s at 8000 rpm, with a Precellys homogenizer (Bertin, France) using the extraction protocol by [76], with a total of 3 mL methanol, 6 mL chloroform and a subsequent washing step with 4 mL water. Net total lipid amount was determined gravimetrically.

To determine lipid composition, lyophilized biomass was extracted with a Soxhlet apparatus using CHCl_3_-hexane (2:1 v/v). The extract was evaporated under N_2_ and weighted and resuspended in hexane. Fatty acid methyl esters (FAME) were obtained, after addition of an aliquot of the internal standard heptadecanoate, by treatment with MeOH-BF_3_ according to the method described by [77]. The quantitative determination of FAME was obtained by gas chromatography using a flame ionization detector (FID-GC). A ZB5-MS 30-m column was used with the following temperature program: 60 °C for 1 min, then an increasing rate of 10 °C min^−1^ up to 180 °C, a second increase of 1 °C min^−1^ up to 230 °C, then 15 °C min^−1^ to reach 290 °C. The injector temperature was 280 °C, the detector temperature was 280 °C; the carrier gas was He with a flow rate of 1 mL min^−1^; splitless injection mode. Based on internal standard area, FAME from FFA, MAG, DAG and TAG were quantitatively estimated on an algae dry weight basis. Compounds were identified by both retention times, comparison of pure standards and gas chromatography coupled to mass spectrometry (GC–MS). Carrier gas was He with a constant flow of 1 mL min^−1^, transfer line temperature to MSD was 280 °C, ionization energy 70 eV, and full scan range 50–500 *m/z*.

### Determination of antioxidant activity

Free radical scavenging activities of lyophilized algae, grown at an irradiance of 1400 µmol photons m^−2^ s^−1^, were determined using the radical species 2,2′-azinobis(3-ethylbenzothiazoline-6-sulfonic acid) (ABTS^•+^) assay as described by [44]. The reducing activity of ethanolic extracts was assessed using FRAP method [43]. FRAP was freshly prepared by mixing (8:1:1, v/v) 0.3 M acetate buffer (pH 3.6), 10 mM 2,4,6-tripyridyl-s-triazine (TPTZ) and 20 mM FeCl_3_. All data are expressed as μmol Trolox Equivalents (TE) per g of WT alga. All measurements were repeated three times.

### Statistics

Significance analysis was performed using either Student’s *t* test or ANOVA test in GraphPad Prism software. Error bars represent the standard deviation.

## Supplementary information

**Additional file 1: Figure S1.** Screening strategy used to isolate pale-green mutant of C. vulgaris. **Figure S2.** Growth curves of WT and pale-green mutants of C. vulgaris, under autotrophic conditions. **Figure S3.** Immunotitration of major photosynthetic subunits. **Table S1.** Acyl chain composition of lipid fraction from WT and mutants PG-14 and SOR.

## Data Availability

The datasets analyzed during the current study available from the corresponding author on reasonable request.

## Abbreviations

β-CC: β-cyclocitral
Car: carotenoids
Chl: chlorophylls
DCMU: 3-(3,4-dichlorophenyl)-1,1-dimethylurea
dhA: dihydroactinidiolide
DW: dry weight
EL: excess light
EMS: ethyl metanesulfonate
*F*_v_/*F*_m_: maximal quantum yield of PSII
LHCI/II: light-harvesting complex of PSI/II
MDA: malondialdehyde
NPQ: non-photochemical quenching
*P*_max_: maximal photosynthetic rate
PAP: 3-phosphoadenosine-5-P
PAR: photosynthetic active radiation
PBR: photobioreactor
PG: pale green
PSI/II: photosystem I/II
RB: Red Bengal
ROS: reactive oxygen species
SOR: singlet oxygen resistant
WT: wild-type
^1^Chl*: singlet excited state of Chl
^3^Chl*: triplet excited state of Chl
^1^O_2_: singlet oxygen
