# Supplementary material for: Combined resistance to oxidative stress and reduced antenna size enhance light-to-biomass conversion efficiency in Chlorella vulgaris cultures
Source: Biotechnol Biofuels. 2019 Sep 16;12:221. doi: 10.1186/s13068-019-1566-9 (PMC6745798; doi:10.1186/s13068-019-1566-9)
Supplement: Supplementary file 1 — Additional file 1: Figure S1. Screening strategy used to isolate pale-green mutant of C. vulgaris. Figure S2. Growth curves of WT and pale-green mutants of C. vulgaris, under autotrophic conditions. Figure S3. Immunotitration of major photosynthetic subunits. Table S1. Acyl chain composition of lipid fraction from WT and mutants PG-14 and SOR. [file 13068_2019_1566_MOESM1_ESM.docx]

**Additional Results**

**Figure S1**

**
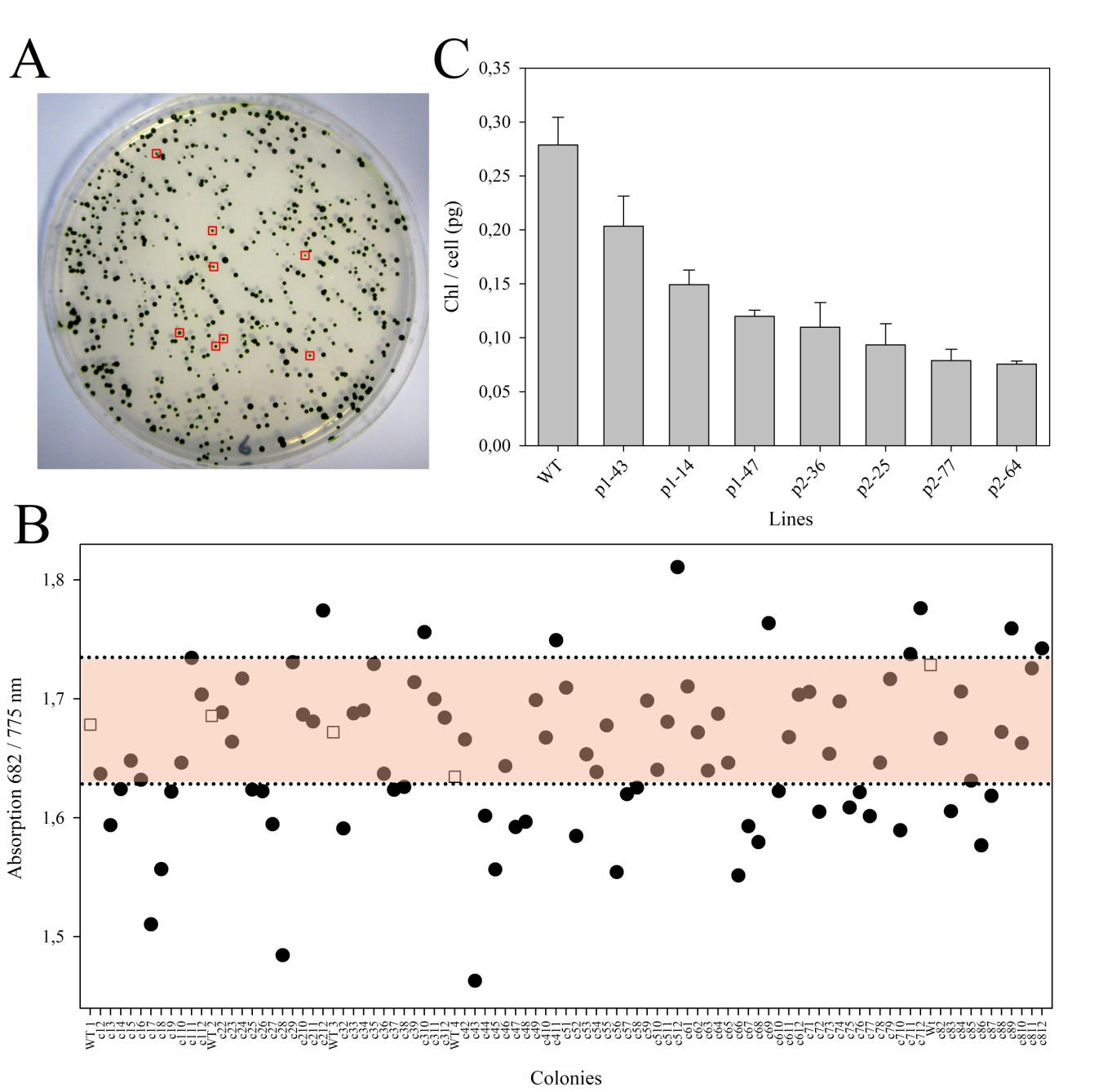
**

**Figure S1. Screening strategy used to isolate pale-green mutant of *C. vulgaris*.** Upon chemical mutagenesis, cells were plated on BG-11 agar plates and exposed to 70 µmol photons m^-2^ s^-1^, 24°C. Single colonies (approximately, 25,000) appeared after 14 days (panel A). Colonies showing a pale-green phenotype were identified by direct sight inspection (90 colonies), then were picked to liquid minimal medium in microtiter plates, grown in the light for seven days, and the Chl content per cell was estimated by measuring absorption of cell suspensions at both 682 and 775 nm (panel B). WT cultures, used as internal standard, showed a 682/775 nm absorption ratio in between 1.63 and 1.73 (rose bar). Strains whose 682/775 ratio was below 1.63 (the lowest values measured for WT) were further selected. Total Chl content analysis was repeated on the most promising lines. This procedure allowed to isolate a group of pale-green mutants (7 lines, panel C).

**Figure S2**

**
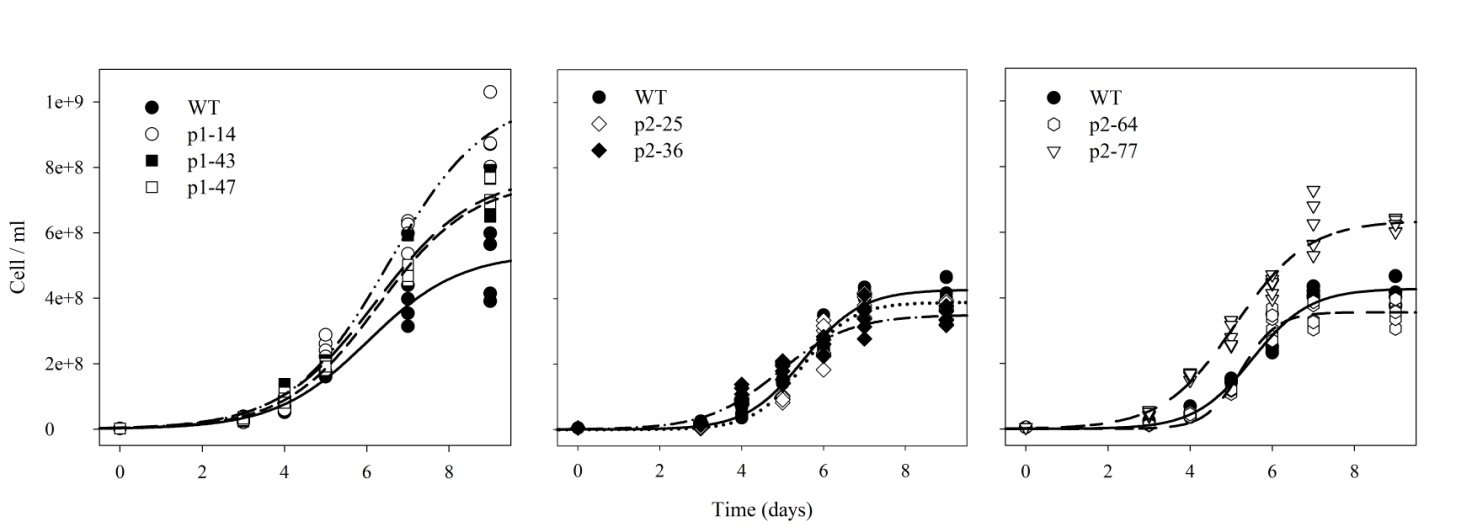
**

**Figure S2.** **Growth curves of WT and pale-green mutants of *C. vulgaris,* under autotrophic conditions.** *C. vulgaris* WT was grown with pale-green mutants and the cell content was measured once a day. All experiments were performed in 1-L cylinders, illuminated with 450 μmol photons m^-2^ s^-1^, 24°C. Growths were performed in a semi-batch system fed with air/CO_2_ mix; the CO_2_ supply was modulated in order to keep the pH of the medium always below 7.1. Each experimental point corresponds to a different sample; data are representative of two independent experiments. Initial inoculum: 1·10^6^ cells ml^-1^. This procedure allowed to identify *PG-14* (isolate p1-14), the pale-green strain with the highest rate of growth, hereafter used.

**Figure S3**

**
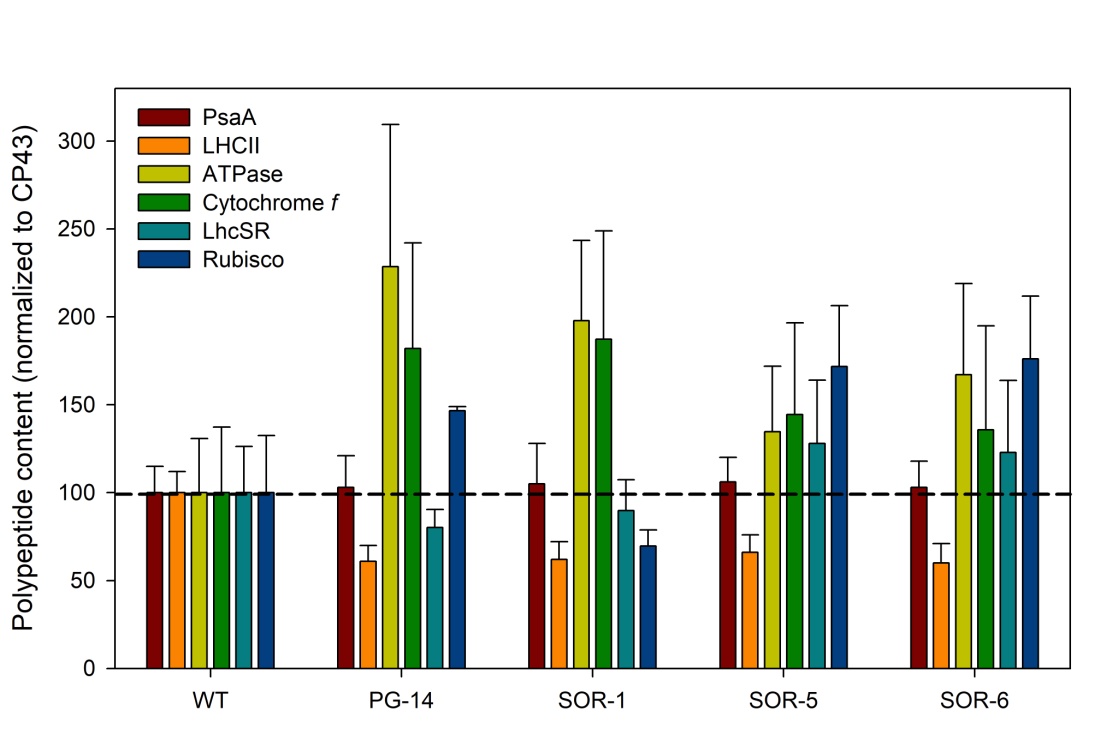
**

**Figure S3. Immunotitration of major photosynthetic subunits.** Total proteins were extracted from WT and mutant cells, grown as reported in Figure 6. Proteins from mutant and control genotypes were fractionated by SDS-PAGE in the same slab gel. The abundance of each subunit in mutants was evaluated by densitometric analysis of western blot, normalized to the PSII core subunit CP43 and expressed as a percentage of the corresponding value in the WT. Data are reported as mean ± SD (n = 3).

**Table S1. Acyl chain composition of lipid fraction from WT and mutants *PG-14* and *SOR*.** Data are expressed as mean ± SD, n = 3. Significantly different values (ANOVA test, *p* < 0.05), within the same column, are marked with different letters.
